# Effect of an Intervention in General Practice to Increase the Participation of Immigrants in Cervical Cancer Screening: A Cluster Randomized Clinical Trial

**DOI:** 10.1001/jamanetworkopen.2020.1903

**Published:** 2020-04-01

**Authors:** Kathy Ainul Møen, Bernadette Kumar, Jannicke Igland, Esperanza Diaz

**Affiliations:** 1 Department of Global Public Health and Primary Care, University of Bergen, Bergen, Norway; 2 Unit for Migration and Health, Norwegian Institute of Public Health (FHI), Oslo, Norway

## Abstract

**Importance:**

Immigrant women have lower participation in cervical cancer screening (CCS) programs. At the same time, some groups of immigrants have higher prevalence of cervical cancer. Targeted interventions are therefore necessary.

**Objective:**

To determine whether an intervention among general practitioners (GPs) could influence immigrant women’s participation in the Norwegian CCS program.

**Design, Setting, and Participants:**

Cluster-randomized clinical trial using the 20 subdistricts of the Bergen, Norway, municipality as clusters. The clusters were matched in 10 pairs according to the number of immigrant women living in them and randomized thereafter. The intervention was implemented between January and June 2017 among urban, primary care, general practices in Bergen. Follow-up ended in January 2018. General practices belonging to the control areas continued treatment as usual. A total of 10 360 women who attended 73 general practices in the 20 subdistricts were included as participants.

**Intervention:**

The intervention consisted of 3 elements: an educational session for GPs at lunch describing the importance of CCS among immigrants and giving advice about how to invite them to participate, a mouse pad as a reminder, and a poster placed in waiting rooms. In the educational session, we elaborated the need for GPs to ask every immigrant woman about CCS, regardless of their reason for contacting their GP.

**Main Outcomes and Measures:**

The main outcome, screening status of immigrant women by January 1, 2018, was obtained from the Norwegian Cancer Registry. The effect of the intervention was measured as odds ratio (OR) for CCS status as of January 1, 2018, for the intervention group vs the control group, with 3 levels of adjustments: baseline CCS status at January 1, 2017 (model 1), additional adjustment for women’s age, marital status, income level, and region of origin (model 2), and further adjustment for the GP’s sex, age, and region of origin (model 3). Two subgroup analyses, screening status at baseline and women’s country of origin, were conducted to assess whether these factors had any influence on the effect of the intervention. Data were analyzed as intention to treat.

**Results:**

A total of 10 360 immigrant women, 5227 (50.4%; mean [SD] age, 44.0 [12.0] years) in the intervention group and 5133 (49.6%; mean [SD] age, 44.5 [11.6] years) in the control group, belonging to 39 general practices in the intervention area and 34 in the control area, were included in the study. The proportion of immigrant women screened increased by 2.6% in the intervention group and 0.6% in the control group. After adjustment for screening status at baseline, women in the intervention group were more likely to have participated in CCS (OR, 1.24 [95% CI, 1.11-1.38]). This statistically significant effect remained unchanged after adjustment for women’s characteristics (OR, 1.24 [95% CI, 1.11-1.38]) and was reduced, but still significant, after further adjustment for GP characteristics (OR, 1.19 [95% CI, 1.06-1.34]). In subgroup analyses, the intervention particularly increased participation among women who were not previously screened at baseline (OR, 1.35 [95% CI, 1.16-1.56]), and those from Poland, Pakistan, and Somalia (OR, 1.74 [95% CI, 1.17-2.61]) when adjusting for baseline screening status.

**Conclusions and Relevance:**

Our intervention targeting general practices significantly increased CCS participation among immigrants, although the absolute effect size of 2% in the fully adjusted model was small. Engaging other primary health professionals such as midwives to perform CCS could further contribute to increasing participation.

**Trial Registration:**

ClinicalTrials.gov Identifier: NCT03155581

## Key Points

**Question:**

Can an educational intervention for general practitioners increase participation in cervical cancer screening among immigrants?

**Findings:**

In this cluster randomized clinical trial that included 73 general practices in 20 clusters, the proportion of immigrant women screened increased by 2.6% in the intervention group and 0.6% in the control group, a significant difference.

**Meaning:**

These findings suggest that increasing general practitioners’ awareness of migrant health issues, such as noncommunicable diseases and lifestyle factors, can improve the health of immigrants.

## Teaser

This cluster randomized trial investigates the effect of an educational intervention for general practitioners on rates of cervical cancer screening among immigrant women in Norway.

## Introduction

Approximately two-thirds of international migrants, some 157 million individuals, resided in high-income countries in 2015.^1^ The influx of migrants to high-income countries from low- and middle-income countries has been increasing in recent years. A report from the World Health Organization^2^ in 2018 called for the inclusion of 2 aspects related to policies aimed at migrant health. The report recommended (1) explicit adoption or application of policies that specifically ensure equity and coverage for various migrant groups and (2) the inclusion of an explicit reference to migrants within general population-based or disease-specific health policies.

Despite the increase in global migration, research in the field of migrants’ health is lagging behind, especially in areas like noncommunicable diseases such as cancer.^3^ Cervical cancer is the fourth most frequent cancer in women. With an estimated 570 000 new cases worldwide in 2018,^4^ cervical cancer ranks second in incidence and mortality after breast cancer in lower-income countries.^5^ Women from sub-Saharan Africa and Southeast Asia have the highest incidence of cervical cancer globally.^5^ One of the reasons for the decrease in incidence and mortality during the last few years in high-income countries could be organized screening programs.^4^

Norway implemented a regular cervical cancer screening (CCS) program based on triennial screening in 1995. Everyone registered in the National Population Registry as a resident in a Norwegian municipality is entitled to have a general practitioner (GP). Asylum seekers and refugees are assigned a provisional identification number on arrival and are thus entitled to a GP as long as their application for asylum has not been rejected. General practitioners have the main responsibility of administering the CCS test, but gynecologists and, to a lesser degree, midwives may also perform the test. All women between ages 25 and 69 years receive a letter of invitation from the Norwegian Cancer Registry, followed by 2 consecutive reminders in the case of nonattendance.

In Norway, 17.8% of the population is either born abroad or born to parents who were born outside Norway.^6^ The participation in the CCS program among immigrants is lower in Norway compared with nonimmigrants,^7,8^ as is the case in other high-income countries.^9,10,11,12,13^ One study^8^ showed that 52% of immigrants and 32% of native Norwegian individuals were nonadherent to CCS.

Barriers to immigrant women’s participation in screening programs have been identified and include challenges not only at the individual level but also at the level of health care practitioners (HCPs) and the health care system.^14,15,16^ However, few studies have described HCPs’ perspectives on CCS among immigrants.^17,18,19^

To our knowledge, no studies to date have investigated the effect of an intervention targeting GPs with a view to increasing participation in CCS among immigrants specifically. This cluster randomized clinical trial evaluates the effect of an intervention targeting general practices with the aim of increasing immigrants’ participation in the regular CCS program in Norway.

## Methods

### Study Design and Participants

**Figure 1.**
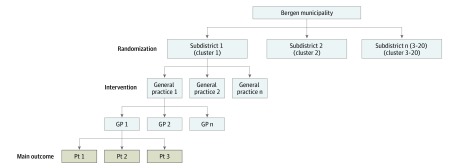
Overview of the Intervention Levels GP indicates general practitioner; and Pt, patient.

This was a community-based, matched-pair, cluster randomized clinical trial. Bergen municipality in western Norway consists of 20 subdistricts, serving as the units of randomization (clusters) in this trial. Women with immigrant backgrounds allocated to the general practices in the clusters are the units of analysis (Figure 1).

All immigrant women and their female offspring (born outside of Norway and Norwegian-born with parents born outside of Norway) who were registered in Norway between January 1, 2012, and January 1, 2018; were between ages 25 and 69 years as of January 1, 2017; and attended a general practice in Bergen as of June 1, 2017, were included in our analysis.

Reporting in this article is aligned with the Consolidated Standards of Reporting Trials (CONSORT) reporting guidelines and the CONSORT Extension for cluster randomized trials. The full trial protocol is available in Supplement 1.

Ethical approval for the trial was obtained from the Regional Ethics Committee of Norway. The researchers did not contact the immigrant women at any time because the information on screening participation was obtained from the Norwegian Cancer Registry. All CCS screening tests are registered in the Norwegian Cancer Registry and are made available for research upon request, but women can request that personal information related to normal findings not be shared by filling out an electronic form. Informed consent from the women was, therefore, not necessary in this study.

### Sample Size

Data for the sample size calculation were obtained from Statistics Norway’s report on immigrants living in Bergen in 2013.^20^ According to the report, the mean (SD) number of immigrant women from low- and-middle income countries aged 20 to 66 years in each of the clusters was 430 (292) and a coefficient of variation for cluster sizes of 0.66. At the time this study was developed, there were no available data on the percentage of immigrant women who participated in the CCS program in Norway; based on international studies^21,22^ and our own proxies,^7^ we conservatively estimated 45% screening participation in the control group. Based on this information and by specifying 5% significance level, the available sample size would be large enough to detect a difference in screening participation of 10 percentage points (45% vs 55%) using a 2-sided 2-sample proportion test with correction for clustering with 80% power as long as the intraclass correlation coefficient did not exceed 0.015.

### Randomization and Masking

All 73 general practices and 232 GPs within the 20 clusters were included in the study. The 20 clusters were matched in 10 pairs according to population size and the percentage of immigrant women from low- and-middle income countries aged 20 to 66 years before randomization, which was the age group available in the report.^20^ Matched-pair randomization was conducted with random allocation sequence generated by using the ralloc command in Stata. General practices were allocated without any allocation concealment or active blinding.

**Figure 2.**
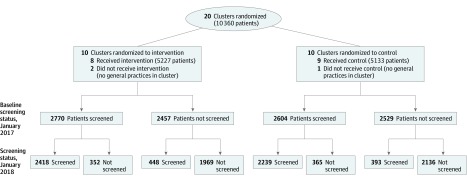
Flowchart of the Intervention

All immigrant women registered with a GP in the 20 clusters were allocated to the intervention or control group. After randomization, 2 clusters from the intervention group and 1 cluster from the control group were excluded because there were no general practices in those clusters. To avoid losing available data, we did not use the matching in the analyses, but chose instead to adjust for characteristics of the different clusters. The exclusion did not result in reduction of sample size because the women living in these 3 excluded clusters had their GPs in 1 of the remaining clusters (Figure 2).

### Intervention Procedure

The intervention was implemented between January and June 2017, and follow-up ended in January 2018. The intervention targeting general practices was based on our previous qualitative study, in which we had focus groups among GPs and semistructured interviews among gynecologists and midwives. They mentioned that there was a lack of knowledge about CCS among HCPs and preferred to enhance that knowledge through short visits from researchers in this field, flyers, and posters that could be placed in the waiting rooms.^17^ The 3 components of the intervention were: (1) a 10- to 15-minute educational session held during the lunch break at the general practices, (2) a mouse pad (eAppendix 2 in Supplement 2) given at the session to remind the GPs of the intervention in their day-to-day work, and (3) a poster to be placed in the waiting rooms (eAppendix 3 in Supplement 2) with the message, “You can prevent cervical cancer with a simple test. Make an appointment with your doctor today!” in Somali, Polish, English, and Urdu. We chose these languages because Polish, Somali, and Pakistani populations represent the largest immigrant groups in Norway. The 10- to 15-minute educational session was theoretically grounded in the review by Chauhan et al^23^ on behavior change interventions among primary care practitioners. The study by Chauhan and colleagues^23^ suggested that interactive, multifaceted medical education programs with collaborative and team-based approaches help to increase screening rates. Our intervention was conducted as a dialogue with the GPs (interactive), had several components (multifaceted), and addressed all the GPs at 1 center together (team-based). The specific content delivered in the educational session is included in eAppendix 1 in Supplement 2.

This educational session was delivered by one of us (K.A.M.), who is a GP. In the educational session, we elaborated on the need for GPs to ask every immigrant woman about CCS, regardless of their reason for contacting their GP.

### Outcomes and Other Measures

Baseline characteristics and follow-up data on the outcome of the study participants were obtained through 3 national registries linked by the unique personal identification number available for each Norwegian resident.

The main outcome measure was screening status as of January 1, 2018. Information on screening status at baseline and following the intervention was obtained from the Norwegian Cancer Registry, where women between ages 25 and 69 years are registered when they take the CCS test.

The demographic data for immigrant women, including age, marital status, highest achieved level of education, income in the calendar year 2016, and region of origin in prespecified subcategories, were obtained from the National Population Registry. Income was categorized in 4 groups according to quartiles of income, based on the distribution of income for all women registered with a GP in Bergen (immigrants and nonimmigrants) as of June 1, 2017. For the women and GPs, region of origin was grouped into European Union (EU)/European Economic Area (EEA), Europe excluding EU/EEA, Africa, Asia (including Turkey), and other countries, as categorized by Statistics Norway. In addition, we had access to women’s country of origin for the biggest immigrant groups. In subgroup analyses, women from the 3 countries who used languages on the poster (Somali, Polish, and Urdu) were studied separately.

The GPs’ age and sex were obtained from the national GP database and the GPs’ region of origin was obtained from the National Population Registry.

### Statistical Analysis

Baseline characteristics of the study population at the individual level (10 360 participants) were reported separately for the intervention and the control group. We also performed descriptive analyses at the cluster level (17 clusters) separately for the intervention group and the control group.

We tested the effect of the intervention using mixed-effects logistic regression with random intercept for subdistrict to account for clustering and reported the intervention effect as odds ratios (ORs) with 95% confidence intervals. In addition, we estimated the absolute difference in proportion of screened women as risk differences using generalized linear models with identity link and binomial distribution with robust clustered standard errors to account for clustering of women within subdistricts. We estimated ORs and risk differences for the intervention effect with 3 levels of adjustment, including variables that were considered as important prognostic factors for the outcome.

In model 1, we adjusted for screening status at baseline. A woman was defined as screened according to Norwegian recommendations if she had undergone CCS testing within 3 years before January 1, 2017 (January 1, 2014, to December 31, 2016). Women who had never been tested or had been tested more than 3 years before January 1, 2017, were defined as not screened at baseline. In model 2, we additionally adjusted for characteristics for the women: age, marital status, education, income level, and region of origin. In model 3, we further adjusted for characteristics of the GPs, including sex, age, and region of origin.

To test whether the effect of the intervention varied in different subgroups, we stratified by screening status at baseline (subgroup analysis 1) and by country of origin, grouping women from 3 countries based on language used in the posters (Somalia, Poland, and Pakistan) vs women from other countries (subgroup analysis 2). We tested for difference in effect between subgroups by including an interaction term between the intervention group and the stratification variable in regression models.

Additionally, we conducted statistical analysis separately for Norwegian-born women with Norwegian-born parents to study the effect of the intervention among Norwegian women living in Bergen in the intervention areas (37 633 women) and control areas (31 636 women) during the same period.

A 5% significance level using 2-sided tests was applied in all analyses, and data were analyzed according to the initial group allocation (intention to treat). We used Stata SE version 15.1 (StataCorp LP) for analysis.

## Results

A total of 10 360 immigrant women were included in the study, with 5227 (50.4%; mean [SD] age, 44.0 [12.0] years) in the intervention group and 5133 (49.6%; mean [SD] age, 44.5 [11.6] years) in the control group. The eTable in Supplement 2 shows baseline characteristics at the cluster level. There were 39 practices in the intervention group and 34 in the control group. All characteristics were similar between the 2 groups, except for the slightly greater number of women, number of GPs, and practices per cluster in the intervention group. This was mainly caused by 1 single cluster in the intervention area (the city center), which was larger in terms of general practices, GPs, and number of women.

Baseline characteristics of the immigrant women are presented for the intervention and control groups (Table 1). Table 2 shows the effect of the intervention in the total sample of women. Intracluster correlation was 0.005 for screening status in January 2018. The last 2 columns show the ORs and the absolute risk differences. In the total study population, the proportion screened according to recommendations had increased from 53.0% (2770 women) to 55.6% (2906 women) in the intervention group, an increase of 2.6%, and from 50.7% (2604 women) to 51.3% (2632 women) in the control group, and increase of 0.6%. After adjustment for screening status at baseline, the OR for being screened at follow-up was 1.24 (95% CI, 1.11-1.38). The effect was the same in model 2 (OR, 1.24 [95% CI, 1.11-1.38]) and remained significant in model 3 (OR, 1,19 [95% CI, 1.06-1.34]). There was a statistically significant absolute risk difference of 2.6% (95% CI, 1.1%-4.0%) between the intervention and control group after adjustment for screening status at baseline that was slightly reduced to 2.0% (95% CI, 0.5%-3.5%) in the fully adjusted model.

**Table 1.** Baseline Characteristics of the Study Population of Immigrant Women in the Bergen Municipality

Characteristic	No. (%)		
	Total	Control	Intervention
No. of women	10 360 (100)	5133 (49.6)	5227 (50.4)
Age, mean (SD), y	NA	44.5 (11.6)	44.0 (12.0)
Marital status			
Single	2479 (23.9)	1011 (19.7)	1468 (28.1)
Married or partner	5835 (56.3)	3101 (60.4)	2734 (52.3)
Seperated, divorced, or widowed	2043 (19.7)	1020 (19.9)	1023 (19.6)
Education			
Primary school	2126 (20.5)	1069 (20.8)	1057 (20.2)
High school or vocational school	2405 (23.2)	1273 (24.8)	1132 (21.7)
University or college	4623 (44.6)	2148 (41.9)	2475 (47.4)
Missing	1206 (11.6)	643 (12.5)	563 (10.8)
Quartiles of income, NOK			
<290 000 (<$30 000)	2748 (26.5)	1269 (24.7)	1479 (28.3)
290 000-689 999 ($30 000-$70 000)	3077 (29.7)	1556 (30.3)	1521 (29.1)
690 000-1 139 999 ($70 000-$120 000)	2914 (28.1)	1549 (30.2)	1365 (26.1)
≥1 140 000 (≥$120 000)	1621 (15.7)	759 (14.8)	862 (16.5)
Region of origin			
European Union or European Economic Area	3556 (34.3)	1843 (35.9)	1713 (32.8)
Europe excluding European Union or European Economic Area	948 (9.2)	515 (10.0)	433 (8.3)
Africa	1218 (11.8)	530 (10.3)	688 (13.2)
Asia, including Turkey	3545 (34.2)	1797 (35.0)	1748 (33.4)
Other	1093 (10.5)	448 (8.7)	645 (12.3)
Cervical cancer screening status at baseline in 2017			
Screened	NA	2604 (50.7)	2770 (53.0)
Not screened	NA	2529 (49.3)	2457 (47.0)

Abbreviations: NA, not applicable; NOK, Norwegian Krone.

**Table 2.** Effect of the Intervention on Participation in CCS in the Total Study Population of 10 360 Immigrant Women in Bergen, Norway

Model	Underwent CCS as of January 2018, No. (%)		OR (95% CI)^a^	RD (95% CI)^b^
	Control (n = 5133)	Intervention (n = 5277)		
Model 1^c^	2632 (51.3)	2906 (55.6)	1.24 (1.11-1.38)	2.6 (1.1-4.0)
Model 2^d^			1.24 (1.11-1.38)	2.5 (1.2-3.9)
Model 3^e^			1.19 (1.06-1.34)	2.0 (0.5-3.5)

Abbreviations: CCS, cervical cancer screening; OR, odds ratio; RD, absolute risk difference.

^a^ Odds ratio for intervention vs control for CCS status in January 2018 (after intervention) estimated using random intercept logistic regression to account for clustering.

^b^ Risk difference for intervention vs control for CCS status estimated using generalized linear model with identity link function and binomial distribution with clustered robust standard errors.

^c^ Adjusted for baseline CCS status (January 2017).

^d^ Adjusted for baseline CCS status (January 2017), woman’s age, marital status, income level, and region of origin.

^e^ Adjusted for all covariates in model 2 plus additional adjustment for general practitioner’s sex, age, and region of origin.

Subgroup analyses by screening status at baseline showed a statistically significant effect in all 3 models for women not screened at baseline (Table 3). The ORs were 1.35 (95% CI, 1.16-1.56), 1.37 (95% CI, 1.18-1.59), and 1.30 (95% CI, 1.11-1.53) for models 1, 2, and 3, respectively. Results for women screened at baseline followed the same trend, but these results were not statistically significant. The test for difference in effect between the 2 subgroups by inclusion of an interaction term was not significant in any of the models.

**Table 3.** Effect of the Intervention on Participation in CCS in Subgroups of Immigrant Women

Subgroup	Underwent CCS as of January 2018, No./total No. (%)		Intervention vs control, OR (95% CI)		
	Control	Intervention	Model 1^a^	Model 2^b^	Model 3^c^
Subgroup analysis 1 by screening status at baseline in 2017					
Not screened	393/2529 (15.5)	488/2457 (19.9)	1.35 (1.16-1.56)	1.37 (1.18-1.59)	1.30 (1.11-1.53)
Screened	2239/2604 (86.0)	2418/2770 (87.3)	1.12 (0.96-1.31)	1.12 (0.96-1.31)	1.08 (0.91-1.28)
*P* value for interaction			.09	.08	.09
Subgroup analysis 2 by country of origin					
Poland, Pakistan, and Somalia	290/824 (35.2)	236/486 (48.6)	1.74 (1.17-2.61)	1.70 (1.12-2.56)	1.54 (0.99-2.40)
Other countries	2342/4309 (54.4)	2670/4741 (57.9)	1.15 (1.02-1.30)	1.16 (1.04-1.31)	1.12 (0.99-1.26)
*P* value for interaction			.02	.01	.01

Abbreviations: CCS, cervical cancer screening; OR, odds ratio.

^a^ Random intercept logistic regression adjusted for baseline CCS status in January 2017 for the total sample and in analyses stratified by country of origin. No adjustment in analyses stratified by screening status at baseline.

^b^ Random intercept logistic regression adjusted for woman’s age, marital status, income level, and region of origin in analyses stratified by screening status at baseline and additional adjustment for baseline CCS status in analyses stratified by country of origin.

^c^ Random intercept logistic regression adjusted for all covariates in model 2 plus additional adjustment for general practitioner’s sex, age, and region of origin.

In subgroup analyses by country of origin (Table 3), the proportion screened among women from Poland, Somalia, and Pakistan at baseline varied between the intervention and the control groups (44.7% vs 35.0%). This was mainly caused by different screening participation of women who belonged to different subdistricts. The majority of the Polish women belonged to 2 subdistricts in the control group with particularly low screening participation at baseline. One subdistrict in the intervention group had high screening participation at baseline. The ORs for subgroup analysis by country of origin with adjustment for screening status at baseline were 1.74 (95% CI, 1.17-2.61) for women from Poland, Somalia, and Pakistan and 1.15 (95% CI, 1.02-1.30) for the rest of the women. The results were similar in the 3 models, although not statistically significant in model 3. *P* values for interactions for test of different intervention effect between the 2 subgroups were statistically significant for all 3 models. The absolute effect size among women from Poland, Somalia, and Pakistan measured as risk differences after adjustment for screening status at baseline was 6.5% (95% CI, 1.8%-11.1%).

Additional analyses among the Norwegian women not included in the main study population revealed an increase in the proportion screened from 64.1% to 65.5% in the control group and an increase from 64.7% to 67.1% in the intervention group. The OR for the intervention effect was 1.03 (95% CI, 0.96-1.10) after adjustment for screening status at baseline; thus, this result was not statistically significant.

## Discussion

This cluster randomized clinical trial among GPs in Norway significantly increased participation of immigrant women in CCS, especially among those who were not previously screened at baseline.

To our knowledge, the present study is the first randomized clinical trial in general practice aiming to increase participation of immigrant women in CCS, and it proved to be effective. Our approach concurs with a recent systematic review and meta-analysis on CCS^24^ stating that interventions based on clinician recommendation and on creating reminder mechanisms for clinicians to initiate testing during opportunistic encounters in the health care setting were effective. However, this review was not specifically based on data from immigrant women. A recent scoping review in Canada^15^ also concluded that HCPs who recommend CCS and take time to explain and listen to immigrant women may increase participation. Two interventions targeting HCPs with a view of boosting screening for cervical and colorectal cancer in the general population have been tested previously. These studies concluded that reminders and recall systems,^25^ through clinician assessment and feedback,^26^ increased participation in CCS.

The intervention showed an effect of 2% in the fully adjusted model. This effect size was significant, but smaller than what we expected to achieve. However, despite the small statistical effect, the clinical significance of this RCT is meaningful, especially because we reached groups who usually have lower participation and are otherwise difficult to reach.

Previous studies suggest that economic restraints of immigrant women and GPs’ time constraints are important barriers to screening,^15^ and our intervention did not change any of these factors. Therefore, in addition to encouraging GPs through targeted interventions such as ours, the inclusion of other HCPs such as midwives in CCS routines could contribute toward even higher participation in the CCS program among immigrants. Immigrant women in Norway already use health clinics where midwives are present for antenatal care and health checkups for their children. These services are free of charge; midwives are mostly female, and they do not have the same time constraints as GPs for their patients.^17^ However, in Norway, midwives neither administer CCS tests routinely nor have the logistics in place to follow up the results. As these are prerequisites for an eventual implementation of the midwives administering the CCS test, a pilot study of such an intervention should be carefully planned and evaluated.

The effect of the intervention was larger among immigrant women who were not screened at baseline, although the interaction test between intervention group and screening status was not significant. This finding concurs with another intervention study for the nonimmigrant population,^27^ consisting of providing mailing lists to the GPs of women who were invited to receive testing but did not respond and inviting women to visit the GP at the office. This intervention resulted in a 6.7% increase in the proportion of previous nonresponders taking the CCS test, which is similar to our results.

Although the effect was statistically significant for all women, irrespective of their country of origin, there was an interaction between country of origin and intervention group, pointing to a larger effect in women from Somalia, Poland, and Pakistan. Ideally, we should have done analyses separately for these 3 countries, but, unfortunately, that was not possible owing to the low number of women from Somalia and Pakistan. Despite this group’s obvious heterogeneity, we chose to group them together because the languages used by these women were used on the poster. After adjustment for differences in baseline screening status, the absolute effect size was 6.5% among these women. The stronger effect related to women from Poland, Somalia, and Pakistan might have resulted not only from women reading the poster, but also because their GPs recommended the CCS test to them more often than to other immigrant groups. Our previous study^17^ and another study from Canada^28^ show that the threshold for HCPs raising the topic of CCS with immigrant women is even higher when the women’s appearance or dress differs from that of the general population. This may be the case for women from Somalia and Pakistan, and our pointing it out to GPs in the intervention during the educational sessions may have contributed to changing the GPs’ attitudes toward these particular groups.

Additional analyses of 69 269 nonimmigrant women living in the same subdistricts of Bergen during the same period, most of whom could probably read the message in English, did not reveal any impact of the intervention among them. Thus, we assume that the effect observed among immigrants was caused by our intervention among GPs that targeted only immigrant women.

### Implications for Future Research

Our study was mainly based on 1 short visit, and we measured the outcome up to 1 year after the intervention. Achieving long-term behavioral change of HCPs and thus obtaining long-lasting effects of health interventions might be challenging after only a single visit. In their hypothesis regarding the promotion of professional behavioral change in health care, Johnson and May^29^ suggested that multifaceted interventions that seek to restructure and reinforce new practice norms are more likely to lead to behavior change. Two of the examples they mention are continuous educational outreach and regular reminders. A future study to measure the effect of this intervention after some years of reminders would be appropriate as the next step in this project. Cost-effectiveness analyses would also be useful but were beyond the scope of the present study. Furthermore, our intervention among GPs might also be relevant for other providers in primary care in urban settings of high-income countries with lower participation of immigrants in CCS and eventually for other preventive interventions.

### Strengths and Limitations

Our study has several methodological strengths. With a cluster randomized design including whole subdistricts with general practices, we avoided selection bias and as much contamination as possible between GPs in the intervention and control groups. The matched-pair clusters had similar characteristics and the intervention trial was implemented without major errors. The measurements in our intervention were objective behavioral change, rather than change in knowledge or self-reported behavior, as is commonly seen in other studies of CCS. With high-quality national registers linked for all the women in the study area for the period of 2012 to 2018, we ruled out self-reported answers, bias toward desirable answers, and false reporting.

This study also has limitations. The 3 clusters with no general practices should have been eliminated before matching and randomization but were eliminated after randomization. Because of this, we could not take matching into account when analyzing the data. However, an impact of this on the results is unlikely because we adjusted for characteristics that could potentially differ between clusters. Owing to a logistical error, 2 practices that should have been allocated to the control group were allocated to the intervention group, and 1 practice that should have been in the intervention group was allocated to the control group. Because it is not possible to identify general practices in the anonymized data set, it was impossible to conduct as-treated analyses to investigate the size of this possible bias. Because of this error, the intervention effect may be slightly underestimated in the conducted intention-to-treat analyses.

## Conclusions

This study found that raising the awareness of GPs and drawing attention to the importance of inviting immigrant women to participate in CCS is a feasible and effective strategy to increase participation in the program, especially among immigrant women who have never been screened. To achieve even greater participation, we suggest studying the effect of regular reminders specifically targeting immigrants in general practice and evaluating the feasibility and effectiveness of including CCS as a task assigned to midwives as a supplemental option to screening done by GPs.

## Notes

Supplement 1. **Trial Protocol**

Supplement 2. **eTable.** Characteristics of Study Population, Analyzed at Cluster Level **eAppendix 1.** The Educational Session Delivered to GPs at the General Practices **eAppendix 2.** Mouse Pad Delivered to Every GP in the Intervention Area as a Reminder **eAppendix 3.** Poster Delivered to General Practices in the Intervention Areas, the Message Was in Somali, Polish, English and Urdu

Supplement 3. **Data Sharing Statement**
