# Supplementary material for: Effect of an Intervention in General Practice to Increase the Participation of Immigrants in Cervical Cancer Screening: A Cluster Randomized Clinical Trial
Source: JAMA Netw Open. 2020 Apr 1;3(4):e201903. doi: 10.1001/jamanetworkopen.2020.1903 (PMC7113727; doi:10.1001/jamanetworkopen.2020.1903)
Supplement: Supplement 1. — Trial Protocol [file jamanetwopen-3-e201903-s001.pdf]

# The trial protocol

## Title: A community-based health intervention trial to increase attendance to the national cervical cancer screening program among immigrants in Norway.

## 1. Relevance relative to the call for proposals

Equity is a cornerstone of the Norwegian health care system. However, there is evidence that there are barriers for immigrant women to access cancer screening programs. This in turn contributes to later diagnosis, worse prognosis and higher utilization of resources among immigrant women with cancer. Although health-related interventions should become increasingly diversity-sensitive [1], adapting services to the individual cultural and religious background is still necessary to reduce health inequalities and provide cost-effective health care and is dependent on the active involvement of users.

In this proposal we present a community-based health intervention trial aimed at increasing participation of immigrant women to the existing cervical cancer-screening program. After a review of the literature and relying on analyses conducted by the research group identifying lower attendants to cervical cancer screening in Norway [2], women from Somalia and Pakistan were selected for the intervention. For this project, we will first invite immigrants from Somalia and Pakistan to identify barriers and factors that influence their attendance to screening of cervical cancer in Norway; then, together with the stakeholders we will develop an adapted intervention based on the existing literature and identified factors; this intervention will be subsequently implemented among women from Somalia and Pakistan and then evaluated using both linked Norwegian registries and qualitative process evaluation. Health-economic analyses will also be conducted.

This community-based study has a parallel trial financed by the Norwegian Cancer Society (2015-2019) with the same aim but targeting health personal that will be conducted in another region in Norway. Taking advantage of this, we propose to conduct a systematic comparison of both interventions. Our health economic analyses will assess the acceptability, equity- and efficiency implications of both interventions. Thus, our proposal will provide new practice-based knowledge and a firmer evidence base to improve public health through the development of health prevention interventions with potential for national implementation within the most common cancer-screening program in Norway. Our design incorporates user involvement from the very beginning of the project and through several stages. It includes an interdisciplinary project group and builds upon cross-sectorial cooperation.

## 2. Aspects relating to the research project

### A. Background and status of knowledge

#### *A.1 Immigrants in Norway*

Over the last decade, there has been a rapid increase in international migration to Norway. Immigrants comprise around 16% of the total population of Norway in 2016. They come from 223 different countries and regions and are younger than Norwegian-born, with over 50% being aged between 20 and 40 years. There are immigrants in all Norwegian municipalities, with higher percentages in Oslo, where immigrants and their descendants make up 32,5 % of the population. Among non-European immigrants, the major immigrant group comes from Somalia, followed by Iraq, Philippines and Pakistan. Immigrants from Somalia are mostly refugees who have lived in Norway for a relatively short period of time, while those from Pakistan have lived in Norway for a long time and have the highest number of descendants in Norway [3].

#### *A.2 Immigration and cancer*

The incidence rates for most cancers are low in developing countries, but for a few cancers, including cervical cancer, the rates are high [4]. Migration studies have found that the risk of cancer change when people move, especially when they move from low-incidence to high-

incidence countries [5]. As migrant populations represent a minority in the host country, unless the host country specifically monitors and targets cancer risk among minority groups, important health care needs among these new immigrants can easily be overlooked [6].

Women comprise about 50% of the global international migrants (95 million) [7]. However, only recently have policy and decision makers started to acknowledge the complexity of the immigrant health and the risks that migrant women can face in the receiving countries [7, 8]. The health issues in immigrants are complex, and a better understanding of cancer in immigrants to Europe and in particular changes in cervical cancer risks is necessary [9, 10] to develop preventive strategies.

### *A.2.1 The Norwegian Cervical Cancer Screening Program*

The Norwegian Cervical Cancer Screening Program (NCCSP) aims to reduce the incidence and mortality of cervical cancer through identifying and treating early cancer stages, which if left untreated have a high likelihood of progressing to invasive cancer. It was implemented in 1995, and it is based on triennial screening with cytology smears among women aged 25-69. Women with no registered screening test during the last three years receive a reminder letter from the Norwegian Cancer Register with a recommendation to make an appointment with their general Practitioner (GP) to have a smear. Overall, 76% of the women have taken a smear after two reminders [11]. While the letters and reminders raise the attendance for the general population, their effectiveness among immigrants is unknown. The screening programme in Norway does not explicitly target immigrant women and hence they are usually under-recruited and not represented at the population level. Yet another constraint is that the reminders are sent in Norwegian, which might dissuade immigrant women from participating in screening or health intervention programmes. According to the Cancer Registry of Norway, more than half of the women diagnosed with cervical cancer have rarely or never taken a Pap test. The NCCSP is changing the test stepwise in Norway, but the type of test taken by the health professional will not affect the results of our study.

### *A.2.2 Immigration and cervical cancer screening*

Cervical cancer is preventable and has a good prognosis if detected early. Although cervical screening has reduced the incidence in many high-income countries, the incidence remains high in countries that do not screen [12]. In accordance to other European countries pointing to low attendance among immigrants, and challenges to access at both the user level and the host health system level [13-15], our research group has newly stated for the first time in Norway that immigrant women, especially from Asia and Africa, are underrepresented in the smears taken at the GP office in Norway [2]. Low attendance to screening programs in turn can result in higher occurrence of cervical cancer in immigrant groups [14] and poor prognoses due to diagnosis at a late stage of cancer, otherwise preventable.

From the *users' point of view*, participation in cervical cancer screening [16] might be influenced by barriers such as fatalistic attitudes, lack of knowledge, fear of cytology smears threatening one's virginity, beliefs that a cytology smear is unnecessary unless one is ill or has risky sexual behaviours, as well as stigmatization within their community and providers [13]. Within the *host health system* barriers such as lack of adequate information for users, language, difficulties in access to health care and varying cultural competence among health workers may prevent new immigrants from following the screening guidelines [17]. However, barriers probably vary among different immigrant groups and in relation to the host country. Therefore, a better understanding of potential cultural, social and other structural barriers to cervical cancer screening programs for different groups of immigrant women in Norway is an essential prerequisite to design culturally appropriate health promotion strategies and screening interventions. The solutions that address these barriers could effectively promote better awareness and encourage women to attend screening, thus diminishing inequity in service utilisation compared to the native Norwegian population.

In this regard, one strategy that has been found to increase cancer-screening participation is by engaging health providers to motivate and directly communicate with women [18, 19]. At the user level, the use of "promoters" (female educators specially trained in the community) has been positively evaluated although the effect to change behaviours has not been evaluated at the population level [20]. Reflecting on these observations, this project will develop and compare two interventions. One intervention involving providers (health personnel) is already under development, financed by the Norwegian Cancer Society. The other one, that we apply funding for, will involve the users (immigrant women), to increase their participation in the cervical cancer screening program in Norway.

## B. Approaches, hypotheses and choice of method

### *B.1. Approaches and hypothesis*

Individual lifestyle factors are influenced by social and community networks, as well as general socio-economic, cultural and environmental conditions [21]. There are several bricks that have to be in place to attain high attendance to screening tests among immigrants. From a *community point of view*, not only women individually need to be aware of the availability of the tests and their benefits and pitfalls, but also the opinion of the other members of the community about taking the tests and the associated barriers to access. Regarding *health practitioners*, they should especially offer the test to the groups with low attendance to screening and high cancer risk, as is the case with some immigrant groups, and to know how to do this in a culturally appropriate way.

Our primary hypothesis is that well developed user targeted and health professional targeted community-level interventions will increase cervical cancer screening attendance rates among immigrant women. Although strategies targeting users and health professionals are thought to be complementary [22], no study to date has compared the effect of each of the approaches to increase participation to screening of cervical cancer. Furthermore, no study to date compares the cost-effectiveness and equity implications of user targeted and health professional targeted interventions for immigrant populations in Norway.

### *B.2. Goals and objectives*

This study is composed by four work packages (WP) of which we apply for founding for three of them (WP1, 3 and 4) while WP2 is already running, financed by the Norwegian Cancer Society (2015-2019).

WP1- *User-targeted* intervention study in Akershus and Buskerud designed to evaluate the effect of a community-based intervention targeting women from Somalia and Pakistan to increase their attendance to the national cervical cancer screening prevention program. The effect will be measured both quantitatively by means of linked national registries and qualitatively through focus groups and in depth interviews.

WP2- *Health professional-targeted* intervention study in Hordaland designed to evaluate the effect of a community-based intervention targeting GPs, midwifes and private gynaecologists to increase their awareness and knowledge about immigrants and cervical cancer screening. The effect will be measured both quantitatively and qualitatively.

WP3- *Comparison of interventions*. To compare the effect of the interventions described above (WP1 and WP2) measured quantitatively (effect size) and qualitatively (how the interventions work). In this way, we will create evidence to guide priority setting.

WP 4- *Cost-analyses* of each of the interventions (WP1 and WP2) will be performed, and cost-effectiveness considered by calculating incremental costs- incremental effectiveness and incremental cost-effectiveness. We will consider equity impact by assessing the distributional consequences for health care utilisation.

### *B.3. Methods*

This study is designed as a community-based health intervention trial. A key feature of such trials is the allocation of intact communities or clusters of individuals rather than individuals themselves to intervention or control groups [23].

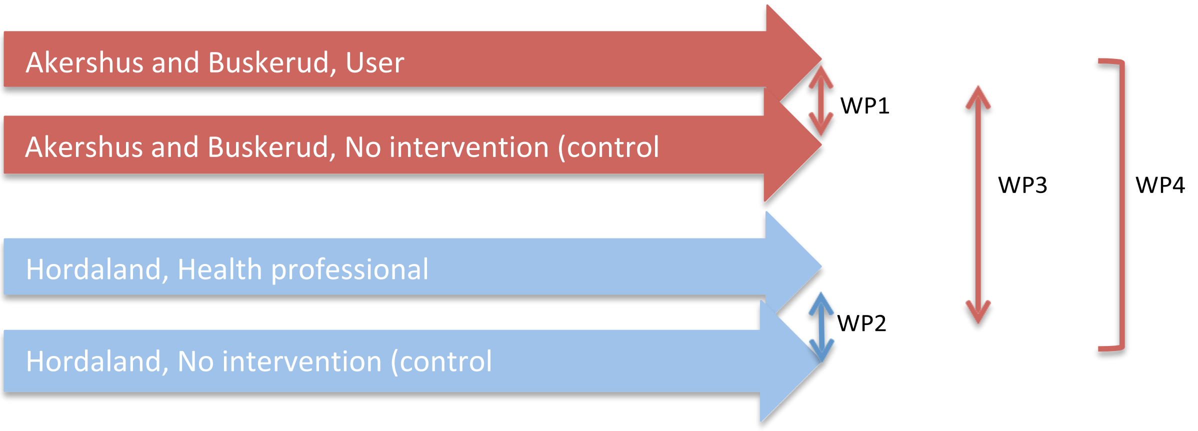


***B.3.1. Interventions, participants and participating areas***

Two different interventions will be developed, one of them meant to increase awareness and attendance among users (WP1), and the other one targeting health professionals (WP2).

*<u>WP1- User intervention</u>*

<u>Assessment previous to the development of the intervention (Timeline 2017/1)</u>

Our approach to the development of the intervention follows the consensus in the field of immigrant health on the need to involve minority ethnic communities in the research process and to support them to define appropriate participatory practices for themselves. Participatory approaches that empower people from these populations, both to define research questions, identify effective interventions and define best practice are considered important to making studies meaningful and effective for tackling inequalities [24]. Thus, the User intervention will be developed based on a) existing literature, b) results from analyses of use of primary care services for screening purposes among immigrants previous to the intervention conducted newly by the study group [2] and c) preliminary interviews with the users (women from Somalia and Pakistan).

Through focus group (FGD) and in-depth interviews including approximately 50 women from Pakistan and Somalia we will explore 1) factors responsible for the low participation rate in the screening program by immigrant women; 2) women's own thoughts about participation in the screening program and how they can help to increase the participation; 3) preferred type of intervention to raise their awareness and participation rate. The participants will be recruited using a maximum variation sampling aimed at diversity in terms of education, age, and number of years lived in Norway. Six to eight FGDs, with each FGD involving six women will be conducted from the two communities separately. Pre-trained females from the communities who are well connected and integrated into local communities will do the recruitment from Pakistan and Somalia.

<u>Participants</u>

Women from Somalia and Pakistan are chosen as targets for the *User intervention* following demographic patterns and earlier analyses [2]. All women from these countries living in the intervention areas compose the intervention groups; immigrant women from the same countries of origin living in control areas will be control participants. It is conservatively estimated that 625 and 915 women in screening age from Somalia and Pakistan respectively live in the study area. Approximately half of each part of the women from each country will live in the intervention clusters and will be invited to the study by the bilingual key women through different channels (invitation through the mosque in collaboration with local stakeholders, information at the local shopping centres, snow-ball oral information, etc).

Area and sample size

This intervention will be implemented in Akershus and Buskerud. These counties are chosen based on the existence of enough immigrants but avoiding areas known to have low participation rates due to on-going research programs in other health fields. Sample size is calculated for an increase in cancer screening participation from 0.45 to 0.55 with 80% power and 5% significance level. After trying several intra class correlation (ICC) levels, we decided to divide four municipalities in Akershus and Buskerud (Asker, Bærum, Lørenskog and Drammen) in 16 clusters that were matched according to the calculated number of female immigrants aged 20 to 66 from Somalia and Pakistan. To avoid contamination, after assuring that pairs were not naturally linked by a mosque or other natural known gather centre for these populations, clusters will be randomly assigned to intervention or control groups (matched pair randomization) [25].

Design and development of the intervention (Timeline 2017/2)

As explained above, we cannot outline the detailed intervention at this point, but based on the existing literature, the possibilities are among others: personal meetings with the women organised according to their practical needs and including the videos, interactive material or written information, etc. The intervention material will be designed and developed in close collaboration with the Norwegian Cancer Society and the NCCSP in order to make it adaptable to the national program in case they prove efficient. We will use professional expertise (communication expert) to develop intervention material.

Implementation of intervention (Timeline 2017/3)

Bilingual key women (project assistants) from Somalia and Pakistan will be recruited and trained as peer educators for their respective communities to increase awareness regarding the importance of cervical cancer prevention and to help peers overcome detected barriers and increase attendance to screening. Strategies to achieve these goals will be developed as explained above (Timeline 2017/2).

*WP2- Heath professional intervention (not included in this application)*

A similar process is being used since 2015 to develop the *Heath professional intervention*, but interviewing health professionals. Bergen was divided into 20 clusters according to the 20 geographical subareas defined by Statistics Norway and similar calculations and matched pair randomization to those described above were conducted. All GPs, midwifes and private gynaecologists working in the intervention cluster areas will be assigned to the intervention group; similar health professionals working in the other municipalities will be chosen as controls. This intervention will be implemented in Hordaland (Bergen) in autumn 2016 to increase awareness of the health professionals involved in screening working in the chosen municipalities. No change in "screening as usual" will be implemented for health professionals working in the control areas. Our experience in this process will facilitate work related to WP1.

### *B.3.2 Measurements*

The study will rely on both quantitative and qualitative evaluation of the interventions in addition to health economic evaluation.

1. *Data from linked national registers*

To avoid the shortcomings of self-reports, outcome information on screening attendance for intervention and controls used for all WPs will be obtained from the Norwegian Cervical Cancer Screening Program, where all pap smears taken in Norway are registered. The leader of this Register is part of the Reference group for this study. From this Register we will apply for information on women who have taken a test, if it is normal or pathological as well as the date for test for all women in Norway. This information will be linked at the individual level to the Norwegian National Registry including information regarding immigrant background (country of origin, reason for migration and length of stay in Norway) for all 25 to 69 years

old registered in Norway, in addition to other socioeconomic variables (age, education, income level, work status, cluster area for those in intervention and control areas, geographical centrality, civil status, husbands' immigrant background and GP's immigrant background) that will be used to adjust our results. Last, a link of the individual woman to his or her GP will be possible through the GP database (Fastlegedatabase) that also includes GP's age and gender. Information will be linked by Statistics Norway, and data will be stored in a secure server at the University of Bergen (BIOS, Core Facility for Biostatistics and Data Analyses) in an anonymised form available only for the study researchers. The Western Regional Committee for Medical and Health Research Ethics has already approved the linking of these data.

*2. Post-intervention qualitative studies* (Timeline 2018/2)

With a similar recruitment strategy and design to the pre-intervention ones but among participants included in the intervention areas, focus groups/in depth-interviews will be conducted after the intervention is implemented among users to assess the underlying processes determining how communities and individuals change their behaviour or fail to do so.

*3. Cost-effectiveness analyses* (WP4: Timelines 2017/4, 2018/4, 2019/3, 2020/2)

The implementation of the two interventions (user- and health professional targeting) will be costed prospectively during the study period. We will apply the perspective of the health provider, although the time use of volunteer peer support will also be estimated and valued. The observed differences in health seeking behaviour will subsequently translate into differences in screening and case detection rates, which also have cost implications. These will be costed separately based on Diagnoses-related group (DRG) rates. A novel analytical model will be developed to combine the trial data with supplementary information where required, to estimate costs, effectiveness and cost-effectiveness of the two interventions. The model will also be used to assess uncertainty, both overall and for single parameters, and whether the uncertainty has potential to influence policy recommendations.

Equity impact will be considered by combining information about the study outcomes with information about the socio-economic background of the participants. We will utilize both descriptive methods, including quintiles analyses, as well as more analytical approaches that also seek to explain the differences in utilization of cancer screening. The latter include estimation of Gini and concentration indices, i.e. the actual inequity in screening, and parametric decomposition of causal factors.

### *B.3.3 Other methodological issues*

*a. Secular trends*

Secular trends, or changes through time disregarding intervention can be a challenge in this type of study. As our outcome measurements rely on linking existing databases, we will include an extended baseline period (from 2012) with repeated measurements of screening attendance several years previous to intervention to better determine the influence of secular trends.

*b. Studying the process and relation with the community*

In addition to the quantitative outcomes, we will examine the process of how our community-based interventions improve health as this information may be just as critical as evaluating the quantitative outcomes.

## 3. The project plan, project management, organisation and cooperation

This project will take advantage of the on-going collaboration between the Norwegian Minority Health Center (NAKMI) and the Department of Global Public Health and Primary Care at the University of Bergen (UiB), both of which will contribute researchers and facilities. In addition, for the design of the intervention we will collaborate with the NCCSP

and the Norwegian Cancer Society, which has already granted a PhD student (Møen) with one 50% position from 2015 to 2019 in order to develop, implement and evaluate the *Health professional's intervention (WP2)*.

Both NAKMI and UiB are excellent educational environments for researchers and responsible for education of health personnel and dissemination of results to society. NAKMI, headed by its director Bernadette N Kumar, has broad experience in project administration and collaboration with immigrant communities and is the *Project owner*. Esperanza Diaz (UiB and NAKMI) has extensive expertise in register epidemiology and immigrant health studies in addition to being part time GP at Bergen municipality with extensive contacts among GPs in Norway. She will be the *Project manager*. Dr Bjarne Robberstad, at UiB, is an expert in health economy analyses and global health. The cross-sectorial cooperation and the interdisciplinarity of the research group hugely increase the feasibility of the study and contribute to further national network building. International partners from Denmark and UK will serve as reference group to additionally ensure the high international standard and quality of our research.

In order to implement the study, we apply the Norwegian Research Council for:

WP1 and WP3: three year 100% Post-doc position, two 20% Researcher four year positions (with specific competence on immigrants from Somalia and Pakistan), 50% Communication expert position for the development of the intervention and two 50% bilingual female Project assistant positions (health promoters) for two years to implement the fieldwork. These positions will be located at NAKMI. The post-doc researcher will collaborate with the other members in the design, implementation and evaluation of the User intervention, while the Researchers will additionally focus on WP3 (comparison of intervention). Dr Kumar and Dr Diaz will be the main responsible for WPs 1 and 3 respectively. At least 6 articles will be published from WP1 and WP3 (see Timeline).

WP4: three year 100% PhD position located at UiB under Dr Robberstad's main supervision with Diaz and Kumar as co-supervisors. Three articles are expected published from WP4.

Dr Diaz has also the main responsibility for WP2 (with a PhD student already financed by the Norwegian Cancer Society), will be the Principal investigator for the whole project and have the main contact with international partners (research grant two months).

*Project group***:** Together with the two part-time Researchers, Post-doc and PhD student that we apply for, the project group will be composed by Kathy Møen (PhD student already financed), Bernadette N Kumar (NAKMI Director and senior researcher), Esperanza Diaz (Project manager, UiB and NAKMI), Bjarne Robberstad (Senior researcher UiB) and one user's representative (from the Norwegian Cancer Society).

*Reference group*: The Norwegian Directorate of health, the Norwegian Cervical Cancer Screening Program, our three international collaborators (see under) and user representatives from immigrant groups have already attended one reference group meeting in relation to WP2. The Project assistants (2x50%), and representatives from intervention municipalities, the Norwegian Medical and Midwife Associations will be invited to the reference group. Users are otherwise involved through the whole research project.

*International collaborators:* Dr. Allan Krasnik and Dr Marie Nørredam (University of Copenhagen, Denmark) and Dr. Sabu Padmadas (Southampton University, UK) have agreed in collaborating as a reference group.

### ***3.1 Timeline***

In this Timeline we present the whole project, also parts of the WP2 for better understanding (in cursive means not included in this proposal).

2017

1. Pre-intervention focus groups with Somali and Pakistani women (WP1, Paper 1)

2. Development of User strategy, recruitment and training of peer educators in Akershus/Buskerud from Somalia and Pakistan (WP1, Paper 2)
3. Implementation User strategy with Somali and Pakistani women (WP1)
4. Development and registration of cost (WP4)
5. *Post-intervention Health professional interviews in Hordaland (WP2)*

2018

1. Writing papers 1 and 2 (WP1)
2. Post-intervention interviews in Akershus/Buskerud (WP1, Paper 3)
3. *Analyses post-intervention register data in Hordaland (2016-2018): attendance to cytology after the Health professional intervention (WP2)*
4. Cost-effective analyses Health professional intervention (WP4, Paper 1)

2019

1. Analyses post-intervention register data in Akershus/Buskerud (2016-2018): attendance to cytology after the User intervention (WP1, Paper 4)
2. Writing papers 3 and 4 (WP1)
3. Cost-effective analyses User intervention (WP4, Paper 2)
4. Comparison of interventions, qualitative approach (WP3, Paper 1)

2020

1. Comparison of interventions, quantitative approach (WP3, Paper 2)
2. Cost-effective analyses: comparison and priority of interventions (WP4, Paper 3)
3. Dissemination of results to communities, stakeholders and policymakers.

## 4. Key perspectives and compliance with strategic documents

### Compliance with strategic documents

Migration is included for the first time as a Sustainable Development Goal in the global development framework [26].Our study complies to the Norwegian National Strategy Document on Immigrant Health [27] which states the necessity of equity in health care for immigrants in Norway [28] and to the Norwegian Cancer Strategy 2013-2017 aiming to promote better information regarding prevention and early diagnosis, and to improve communication with cancer patients where language may be a barrier [29]. Our intervention design in collaboration with stakeholders addresses the translational gap between evidence and practice [30]. Immigrant community engagement is one of the practice strategies to address delivery challenges for migrants identified by consensus in the field [31].

### Relevance and benefit to society

A more heterogeneous population with more minorities and growing social inequality in health is placing new demands on health services. To achieve high quality service and ensure that the services are perceived as beneficial by those concerned, we will incorporate experiences and views from both users and service providers into the development of our study. Cervical cancer is one of the few preventable cancers, if detected early. It is the third most common cancer and the fourth most frequent cause of cancer deaths in women worldwide [32]. A higher attendance to the cancer-screening program among immigrants will help detect cancer in earlier stadium, when they still can be cured without sequels for the person and with an economical benefit for society at large.

### The project will not have any environmental impact

### Ethical perspectives

The project is already approved by the Norwegian Regional Committees for Medical and Health Research Ethics (REK 2015/1156). A special issue regarding community health-intervention trials is, to which degree the populations involved should be informed about the study. This is especially important concerning the control groups, because their ignorance about the existence of an intervention can be crucial. This issue is approved by REK. Also,

since ethnicity is considered sensitive data, and in order to make the dataset completely anonymous for the research group, we will only apply for immigrant background classified in major groups with regard to country of origin, and use the BIOS system at UiB.

Because we include users in the very development of the interventions, we cannot specify how the intervention will look like, although we can assume that the interventions will probably not be very different of what is described in the literature and will not pose further ethical challenges. All women are already invited to take the screening program through a letter from the NCCSP, although this letter is in Norwegian. The User intervention will be implemented through peer educators and will include culturally adapted messages to women voluntarily attending to meetings. The possibility of having meetings with husbands, brothers or fathers will be discussed in focus groups so that we do not pose any inconvenience to women struggling with gender issues [33].

**Gender issues**

Only women will be included in this study. Because the study theme is for some cultures difficult to disclose with men, all the study members in contact with participants will be women. In addition, we pay special consideration to bilingual researchers and staff.

## 5. Dissemination and communication of results

a) Several per-reviewed scientific publications in international journals and communications at international conferences (WP1: 4 Papers, WP3: 2 Papers, WP4: 3 Papers)
b) Popular scientific dissemination in journals like Utposten and NAKMI's reports and webpage
c) Direct feedback to the study participants in both intervention and control areas at the end of the study
d) Communication to the Heath Directorate, primary health services and local policymakers at specific meetings

**Communication with users**

Users are invited at several stages in this project. They are part of the reference group, collaborate to develop the interventions and evaluate how/if the interventions are working. Additionally, results from the study will be disseminated among participants in intervention and control areas.

## *6. Additional information specifically requested in the call for proposals*

**User involvement**

We will invest time and effort to establish a relationship with the community based on trust, mutual benefit and feedback. The immigrant background and female gender of several senior researchers in this group will probably help us to achieve this goal. Female bilingual project assistants from Somalia and Pakistan will be recruited and they will in turn be in charge of recruitment and contact with their own communities. Users will be part of the development of the intervention and will again be interviewed after the intervention to understand its effect.

**Anticipated benefits**

Through this study, we will provide new practice-based knowledge and a firmer evidence base to improve attendance to a preventive health care intervention and thus contribute to equal health care and good health for society at large- including the immigrant population. Specifically, our project will contribute to: 1) the development of strategies targeting immigrant users that can be used after the project by the national screening program, 2) an increase of awareness on screening and cancer in the intervention groups, 3) enhanced understanding of the health care system for immigrant women in the intervention groups and 4) increase of attendance to cytology and thus earlier recognition of treatable cancer in the

intervention areas, where many immigrants from Somalia and Pakistan live.

## References


1. Razum, O. and J. Spallek, *Addressing health-related interventions to immigrants: migrant-specific or diversity- sensitive?* Int J Public Health, 2014. **59**: p. 893-895.
2. Møen, K.A., et al., *Differences in cervical cancer screening between immigrants and non- immigrants in Norway – A primary health Care register-based study.* Submitted 2016.
3. Statistics Norway, S., *Key figures for immigration and immigrants*. 2016.
4. Hemminki, K., et al., *Cancer in immigrants as a pointer to the causes of cancer.* Eur J Public Health, 2014. **24 Suppl 1**: p. 64-71.
5. Ziegler, R.G., et al., *Migration patterns and breast cancer risk in Asian-American women.* J Natl Cancer Inst, 1993. **85**(22): p. 1819-27.
6. Hien, A. and J. Lafontant, *[Inequities in health in minority communities: diagnosis of the situation among the Francophone immigrants of Sudbury].* Can J Public Health, 2013. **104**(6 Suppl 1): p. S75-8.
7. United Nations Population Fund., *State of world population 2006. A passage to hope - Women and international migration.* 2006: NewYork.
8. World Migration Report 2005. *Costs and Benefits of International Migration*. Available from: http://publications.iom.int/bookstore/index.php?main_page=popup_image&pID=176&zenid=0p7hdao39bf14ld4hg_5n9ro4l4.
9. Geddes M, et al., *Cancer in Italian Migrants Populations.* IARC Scientific Publication No. 123, 1993.
10. Azerkan, F., et al., *Risk of cervical cancer among immigrants by age at immigration and follow-up time in Sweden, from 1968 to 2004.* Int J Cancer, 2008. **123**(11): p. 2664-70.
11. Lönnberg S and S. G., *Masseundersøkelsen mot livmorhalskreft. Årsrapport 2009-2011.* . 2013, Kreftregisteret: Oslo.
12. IARC, *Cervix cancer screening*. 2005, Working Group on the Evaluation of Working Group on the Evaluation of Cancer Preventive Strategies. : Lyon.
13. McDonald, J.T. and S. Kennedy, *Cervical cancer screening by immigrant and minority women in Canada.* J Immigr Minor Health, 2007. **9**(4): p. 323-34.
14. Azerkan, F., et al., *Cervical screening participation and risk among Swedish-born and immigrant women in Sweden.* Int J Cancer, 2012. **130**(4): p. 937-47.
15. Latif, E., *Recent immigrants and the use of cervical cancer screening test in Canada.* J Immigr Minor Health, 2010. **12**(1): p. 1-17.
16. Johnson, C.E., et al., *Cervical cancer screening among immigrants and ethnic minorities: a systematic review using the Health Belief Model.* J Low Genit Tract Dis, 2008. **12**(3): p. 232-41.
17. Scarinci, I.C., et al., *Cervical cancer prevention: new tools and old barriers.* Cancer, 2010. **116**(11): p. 2531-42.
18. Beydoun, H.A. and M.A. Beydoun, *Predictors of colorectal cancer screening behaviors among average-risk older adults in the United States.* Cancer Causes Control, 2008. **19**(4): p. 339-59.
19. Kwok, C., R. Cant, and G. Sullivan, *Factors associated with mammographic decisions of Chinese-Australian women.* Health Educ Res, 2005. **20**(6): p. 739-47.
20. Luque, J.S., et al., *Salud es vida: development of a cervical cancer education curriculum for promotora outreach with Latina farmworkers in rural Southern Georgia.* Am J Public Health, 2011. **101**(12): p. 2233-5.
21. Dalgren, G., *Opportunities for the Future Vol 1-Intersectorial Action for Health,*, in *European Health Policy Conference.* . 1995: Copenhagen.WHO Regional Office for Europe.
22. Schleicher, E., *Immigrant Women and Cervical Cancer Prevention in the United States.*, W.s.a.C.s.H.P. Center, Editor. 2007: Johns Hopkings Bloomberg Schol of Public Health.
23. Donner, A., *Methodological issues in the design and analysis of community intervention trials*, in *Oxford Textbook of Public Health (5 ed.*, R.B. Roger Detels, Mary Ann Lansang, and Martin Gulliford, Editor. 2011, Oxford University Press.
24. Mir, G., et al., *Principles for research on ethnicity and health: the Leeds Consensus Statement.* European Journal of Public Health, 2012.
25. Atienza, A.A. and A.C. King, *Community-based health intervention trials: an overview of methodological issues.* Epidemiol Rev, 2002. **24**(1): p. 72-9.
26. Migration, I.O.f. *2030 Agenda for Sustainable Development*. 2016 [cited 2016 April 2016]; Available from: https://unobserver.iom.int/2030-agenda-sustainable-development.
27. Helsedirektoratet, *Nasjonal Strategi om Innvandreres helse 2013-2017 (Norwegian National Strategy Document on Immigrant Health)*, M.o. Rehabilitering, Editor. 2012: Oslo.
28. Nissen-Meyer, M., A.L. Shuja, and T. Sletnes, *Likeverdig helsetjeneste? Om tjenester til ikke-vestlige innvandrere*, D.n. legeforening, Editor. 2008.
29. Services, N.M.o.H.a.C., *Together-against cancer. National Cancer Strategy 2013-2017*. 2013.
30. MacFarlane, A., et al., *REsearch into implementation STrategies to support patients of different ORigins and language background in a variety of European primary care settings (RESTORE): study protocol.* Implement Sci, 2012. **7**: p. 111.

31. Pottie, K., et al., *Improving delivery of primary care for vulnerable migrants: Delphi consensus to prioritize innovative practice strategies.* Can Fam Physician, 2014. **60**(1): p. e32-40.
32. Jemal, A., et al., *Global cancer statistics.* CA Cancer J Clin, 2011. **61**(2): p. 69-90.

Thiel de Bocanegra, H., et al., *Mexican immigrant male knowledge and support toward breast and cervical cancer screening.* J Immigr Mino
