# Supplementary material for: Effect of an Intervention in General Practice to Increase the Participation of Immigrants in Cervical Cancer Screening: A Cluster Randomized Clinical Trial
Source: JAMA Netw Open. 2020 Apr 1;3(4):e201903. doi: 10.1001/jamanetworkopen.2020.1903 (PMC7113727; doi:10.1001/jamanetworkopen.2020.1903)
Supplement: Supplement 2. — eTable. Characteristics of Study Population, Analyzed at Cluster Level eAppendix 1. The Educational Session Delivered to GPs at the General Practices eAppendix 2. Mouse Pad Delivered to Every GP in the Intervention Area as a Reminder eAppendix 3. Poster Delivered to General Practices in the Intervention Areas, the Message Was in Somali, Polish, English and Urdu [file jamanetwopen-3-e201903-s002.pdf]

Supplementary Online Content

Møen KA, Kumar B, Igland J, Diaz E. Effect of an intervention in general practice to increase the participation of immigrants in cervical cancer screening: a cluster randomized clinical trial. *JAMA Netw Open*. 2020;3(4):e201903. doi:10.1001/jamanetworkopen.2020.1903

eTable. Characteristics of Study Population, Analyzed at Cluster Level

eAppendix 1. The Educational Session Delivered to GPs at the General Practices

eAppendix 2. Mouse Pad Delivered to Every GP in the Intervention Area as a Reminder

eAppendix 3. Poster Delivered to General Practices in the Intervention Areas, the Message Was in Somali, Polish, English and Urdu

This supplementary material has been provided by the authors to give readers additional information about their work.

© 2020 Møen KA et al. *JAMA Network Open.*

eTable: Characteristics of study population, analyzed at cluster level

	Usual Care	Intervention
Nr of clusters, n	9	8
Number of GP-practices, n	34	39
Number of GP's, n	104	128
Number of GP-practices per cluster, median (min-max)	3 (1-8)	3.5 (1-16)
Number of GPs per cluster, median (min-max)	9 (4-23)	13.5 (1-45)
Number of women per cluster, mean (SD)	570 (411)	653 (708)
Mean age in clusters, mean (SD)	45.0 (2.2)	44.9 (1.5)
Proportion married, mean (SD)	0.6 (0.07)	0.6 (0.08)
Proportion with university/college education, mean (SD)	0.5 (0.09)	0.5(0.09)
Proportion with income < 290 000 NOK (30 000 Eur), mean (SD)	0.2 (0.08)	0.3 (0.07)

© 2020 Møen KA et al. *JAMA Network Open.*

Proportion from Africa, Middle East, South Asia and South-East Asia, mean (SD)	0.4 (0.09)	0.4 (0.06)
Proportion with CCS status screened at baseline. mean (SD)	0.5 (0.07)	0.5 (0.02)

© 2020 Møen KA et al. *JAMA Network Open.*

eAppendix 1

The educational session delivered to GPs at the general practices

Thank you for giving me the opportunity to talk about cervical cancer among immigrant women. As you know, I am a GP, and take a doctoral thesis on this subject at the University of Bergen.

We know that immigrants are a large group with many different cultures and nationalities, but if you think a bit back: how often do you take cervical cancer tests from immigrants?

What we have found in our research is that the immigrant women, no matter where they come from, have lower attendance to cervical screening than Norwegian-born women. In addition, we know that some immigrant groups, especially those from East Africa and South-East Asia, have a higher incidence of cervical cancer. Therefore, we collaborate on this project with the Norwegian Cancer Registry and the Norwegian Cancer Society, which also believe that immigrant women should be given priority in order to detect cancer as early as possible.

As I said earlier, we know that there are differences between the immigrant groups, but I am generally speaking now because this really concern most immigrant women. Women from Somalia and Pakistan who we interviewed say that they do not receive or do not read the reminder letter from the Cancer Registry.

Those who try to read do not understand the letter. They also say that the doctors did not address the issue with them, but they would really be interested in getting an appointment for the cervical cancer test if they had understood that this was important for them. It is all right that women decide not to take the test if they want, but they must be given relevant information about cervical cancer screening. It is therefore important that we, as GPs, inform immigrant women, briefly and in simple language, that this is a test to avoid cancer in the genital area and this is recommended for all women between the ages of 25 and 70. Since many believe that the test should first be taken when one has symptoms, it is important to emphasize that the test should be taken before getting symptoms and regardless of how many partners one has. The latter is also a common misconception among some immigrant women, that if you have only one partner, you do not have to take the test. Therefore, today we will invite you to a local campaign to try to increase the attendance to cervical screening among immigrant women in general and especially among non-western immigrants.

The campaign has two parts:

1. We have created nice posters that we hope you can hang in the waiting room, in the laboratory or at the doors of their office. The posters are mostly meant for women, so they themselves are more interested in getting information and ordering time. You may want to inform the medical secretaries about the campaign so that they can help with a brief explanation if immigrant women ask about the posters. Our aim is to not overload you, but to offer the women equal health services and that they are offered own appointments for cervical cancer test if they are interested.

2. The other part of the campaign is inviting you to take this topic up more often with the patients when they come for other reasons. When we talked to GPs in connection with the project, a great

number of doctors said that they rarely address the topic of cervical cancer test with women from non-western countries because these patients addressed many issues on one consultation and then it was difficult to make time to take this topic up as well. However, since they rarely take this test and some of them are most susceptible to lethal form of cancer, we suggest that you consider mentioning this to the women in connection with the consultations. It is probably enough to inform, in brief and in simple language, that this is a test to avoid cervical cancer, it is recommended to all women between 25 and 70 and that it should be taken before getting symptoms regardless of how many partners you have. From our own experience, appointments should be preferably given before they leave the office, instead of asking them to make an appointment when it suits.

Our experience after talking to the GPs is that male doctors have a low threshold to offer referrals to female colleagues / gynaecologists to these patients and it is all right if male GPs think that if this the right way, but giving information to women is the most important thing. So: 1. Place the posters 2. ask the women about cervical cancer test, are our main messages.

Thank you for your attention!

eAppendix 2

Mouse pad delivered to every GP in the intervention area as a reminder

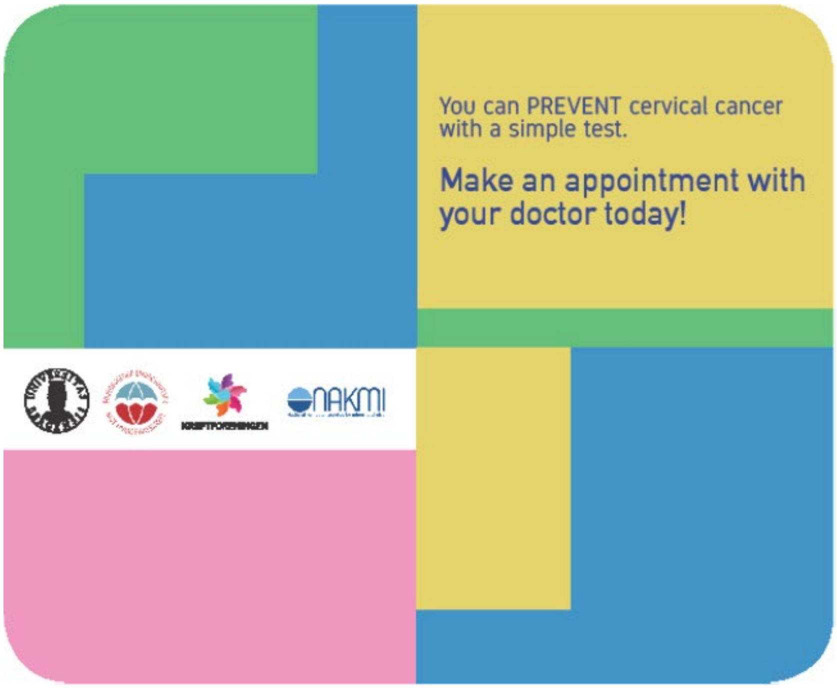

© 2020 Møen KA et al. *JAMA Network Open.*

eAppendix 3

Poster delivered to general practices in the intervention areas, the message was in Somali, Polish, English and Urdu.

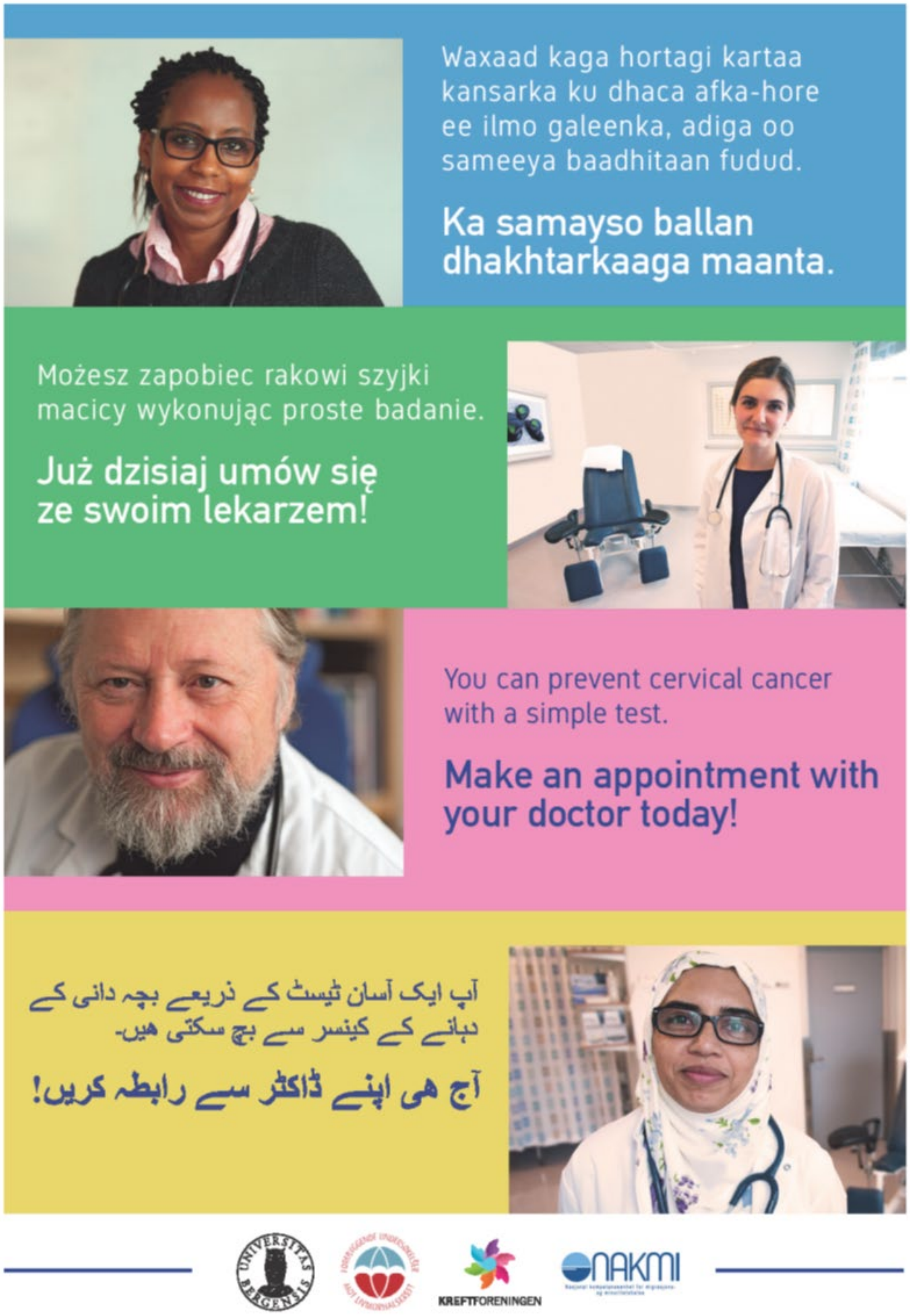

© 2020 Møen KA et al. *JAMA Network Open.*
