# Supplementary material for: Effect of an Intervention in General Practice to Increase the Participation of Immigrants in Cervical Cancer Screening: A Cluster Randomized Clinical Trial
Source: JAMA Netw Open. 2020 Apr 1;3(4):e201903. doi: 10.1001/jamanetworkopen.2020.1903 (PMC7113727; doi:10.1001/jamanetworkopen.2020.1903)
Supplement: Supplement 3. — Data Sharing Statement [file jamanetwopen-3-e201903-s003.pdf]

Data Sharing Statement

Møen KA, Kumar B, Igland J, Diaz E. Effect of an intervention in general practice to increase the participation of immigrants in cervical cancer screening: a cluster randomized clinical trial. *JAMA Netw Open*. 2020;3(4):e201903. doi:10.1001/jamanetworkopen.2020.1903

Data

Data available: Yes
Data types: Deidentified participant data
How to access data: Data will be provided on request by the project's principal investigator, Dr Esperanza Diaz. Contact her at esperanza.diaz@uib.no
When available: With publication

Supporting Documents

Document types: Statistical/analytic code
How to access documents: These supporting documents will be provided on request by the project's principal investigator, Dr Esperanza Diaz. Contact her at esperanza.diaz@uib.no
When available: With publication

Additional Information

Who can access the data: Data will be made available for researchers whose proposed use of the data has been approved.
Types of analyses: Data will be made available for researchers with approved specific purposes.
Mechanisms of data availability: With investigator support, after approval of the proposal and with a signed data access agreement.
Any additional restrictions: The researcher's project should have been approved by a committee of ethics.
